# Intensity harmonization techniques influence radiomics features and radiomics-based predictions in sarcoma patients

**DOI:** 10.1038/s41598-020-72535-0

**Published:** 2020-09-23

**Authors:** Amandine Crombé, Michèle Kind, David Fadli, François Le Loarer, Antoine Italiano, Xavier Buy, Olivier Saut

**Affiliations:** 1 Department of Radiology, Institut Bergonie, 33000 Bordeaux, France; 2 Modelisation in Oncology (MOnc) Team, INRIA Bordeaux-Sud-Ouest, CNRS UMR 5251, Université de Bordeaux, 33405 Talence, France; 3 University of Bordeaux, 33000 Bordeaux, France; 4 Department of Pathology, Institut Bergonie, 33000 Bordeaux, France; 5 Department of Medical Oncology, Institut Bergonie, 33000 Bordeaux, France; 6 Department of Diagnostic and Interventional Radiology, Institut Bergonié, Comprehensive Cancer Center of Nouvelle-Aquitaine, 229 cours de l’Argonne, 33000 Bordeaux, France

**Keywords:** Computational biology and bioinformatics, Biomarkers, Oncology

## Abstract

Intensity harmonization techniques (IHT) are mandatory to homogenize multicentric MRIs before any quantitative analysis because signal intensities (SI) do not have standardized units. Radiomics combine quantification of tumors’ radiological phenotype with machine-learning to improve predictive models, such as metastastic-relapse-free survival (MFS) for sarcoma patients. We post-processed the initial T2-weighted-imaging of 70 sarcoma patients by using 5 IHTs and extracting 45 radiomics features (RFs), namely: classical standardization (IHT_std_), standardization per adipose tissue SIs (IHT_fat_), histogram-matching with a patient histogram (IHT_HM.1_), with the average histogram of the population (IHT_HM.All_) and plus ComBat method (IHT_HM.All.C_), which provided 5 radiomics datasets in addition to the original radiomics dataset without IHT (No-IHT). We found that using IHTs significantly influenced all RFs values (p-values: < 0.0001–0.02). Unsupervised clustering performed on each radiomics dataset showed that only clusters from the No-IHT, IHT_std_, IHT_HM.All_, and IHTHM.All.C datasets significantly correlated with MFS in multivariate Cox models (p = 0.02, 0.007, 0.004 and 0.02, respectively). We built radiomics-based supervised models to predict metastatic relapse at 2-years with a training set of 50 patients. The models performances varied markedly depending on the IHT in the validation set (range of AUROC from 0.688 with IHT_std_ to 0.823 with IHT_HM.1_). Hence, the use of intensity harmonization and the related technique should be carefully detailed in radiomics post-processing pipelines as it can profoundly affect the reproducibility of analyses.

## Introduction

Radiomics has now become an intensive field of research, based on the extraction and mining of several quantitative variables, which are referred to as radiomics features (RFs). RFs enable to screen extensively the shape and texture of objects of interests within medical images of any modality. In oncology, RFs have been used in predictive models based on machine-learning classifiers to discriminate benign and malignant lesions, identify molecular alterations in tumors, predict patients’ outcome, and even build radio-genomics signatures^1–3^. Regarding sarcomas, radiomics have improved predictions of grading, prognosis and response to chemotherapy/radiotherapy, based on CT-scans, structural MRI alone or combined with positron emission tomography, dynamic-contrast enhanced or diffusion MRI^4–9^.

Though one aim of radiomics is to provide an objective assessment of tumor phenotype, several studies have shown the influence of pre- and post-processing factors on the value of RFs^10–15^. These findings question the validity and reproducibility of inter-site radiomics studies. This issue is even more prominent with MRI because of the absence of standard intensity scale. Therefore, signal intensities (SIs) lack of comparability, even for a given sequence acquired on the same MR-scanner. Unlike gray-levels discretization or voxel-size standardization, technical details regarding homogeneization of SIs are frequently missing in materials and methods and, even when performed, assessment of the optimal setting for the MRI dataset of interest is often lacking.

Some intensity harmonization techniques (IHTs) have been proposed in the neuroimaging literature to enable robust analysis of structural and diffusion MRIs across different radiological centers and longitudinally, but most cannot be transposed to sarcomas because of the heterogeneity of tissues surrounding sarcomas, which are ubiquitous tumors. Available IHTs regarding non-brain MRI are scarce. The most frequently encountered are global scaling (e.g. where SIs values are centered by removing the mean and scaled to unit variance, or transformed to range between 0 and 1), ratio with SIs of a healthy tissue that is not affected by the disease (for instance adipose tissue or muscle in musculoskeletal imaging), or histogram-matching (HM, where the intensity histograms are transformed to match a reference intensity histogram)^16–18^. In addition, Orlhac et al. have recently shown that ComBat harmonization method, which was initially described in genomics to remove batch effect, could correct non biological differences related to the type of scanners^19^. Though the authors focused on CT-scanner, ComBat may help reduce unwanted variations in MRI-based radiomics datasets as well.

Thus, our aim was to investigate how the IHT could influence MRI-based radiomics analyses in a uniformly-treated cohort of soft-tissue sarcomas (STS) patients with which the presence of intra-tumor heterogeneity on initial T2-weighted-imaging (-WI) has been previously correlated with metastatic-relapse free survival (MFS)^4,6,20^. To do so, to comprehensively assess the impact of IHT on radiomics analyses, we investigated its influence on: (i) the RFs values; (ii) the prognostic value of radiomics-based unsupervised classifications; and (iii) the performances of supervised classifiers to predict early metastatic relapses.

## Methods

### Study population

This study was approved by the local Research Ethics Committee of Bergonié Insitute (Bordeaux, France) according to good clinical practices and applicable laws and regulations. All methods were performed in accordance with the relevant guidelines and regulations. The need for written informed consent was waived because of its retrospective nature.

Patients were consecutively recruited as they fulfilled the following inclusion criteria: newly-diagnosed, non-metastatic (according to chest CT-scan), histologically-proven high-grade STS of trunk wall or extremities (n = 163), treated with 4–6 cycles of anthracycline-based neoadjuvant chemotherapy and curative surgery at our sarcoma reference center from June 2006 to November 2016 (n = 133), available baseline MRI (n = 95) with axial spin-echo T2-WI without artefacts (n = 72), and available clinical and radiological follow-ups for at least 2 years after the surgery (n = 70). Follow-ups consisted in a clinical examination and chest radiograph every 3 months for 2 years, every 6 months for 5 years and annually until 10 years after surgery, which were complemented by chest CT-scans and MRIs in case of doubtful findings. All relapses were histopathologically confirmed. MFS was defined as the time since curative surgery to metastatic relapse.

### MRI acquisition

The baseline MRI examinations were acquired on 3 different 1.5-T MR-systems (Philips Signa [17/70, 24.3%], Siemens MAGNETOM Aera [41/70, 58.5%], General Electrics Healthcare Optima Jem MR450w [12/70, 17.1%]) with adjustment of coils, field-of-view and matrix depending on tumor size, location and depth. Regarding T2-WI, the range of repetition and echo times were 2,400–4,500 ms and 70–130 ms, respectively. Slice thickness ranged from 3 to 5 mm. The protocol also systematically included 2D or 3D T1-WI after intra-venous gadolinium-chelates injection (with or without fat-suppression).

### MRI post-processing (Fig. 1)

**Figure 1:**
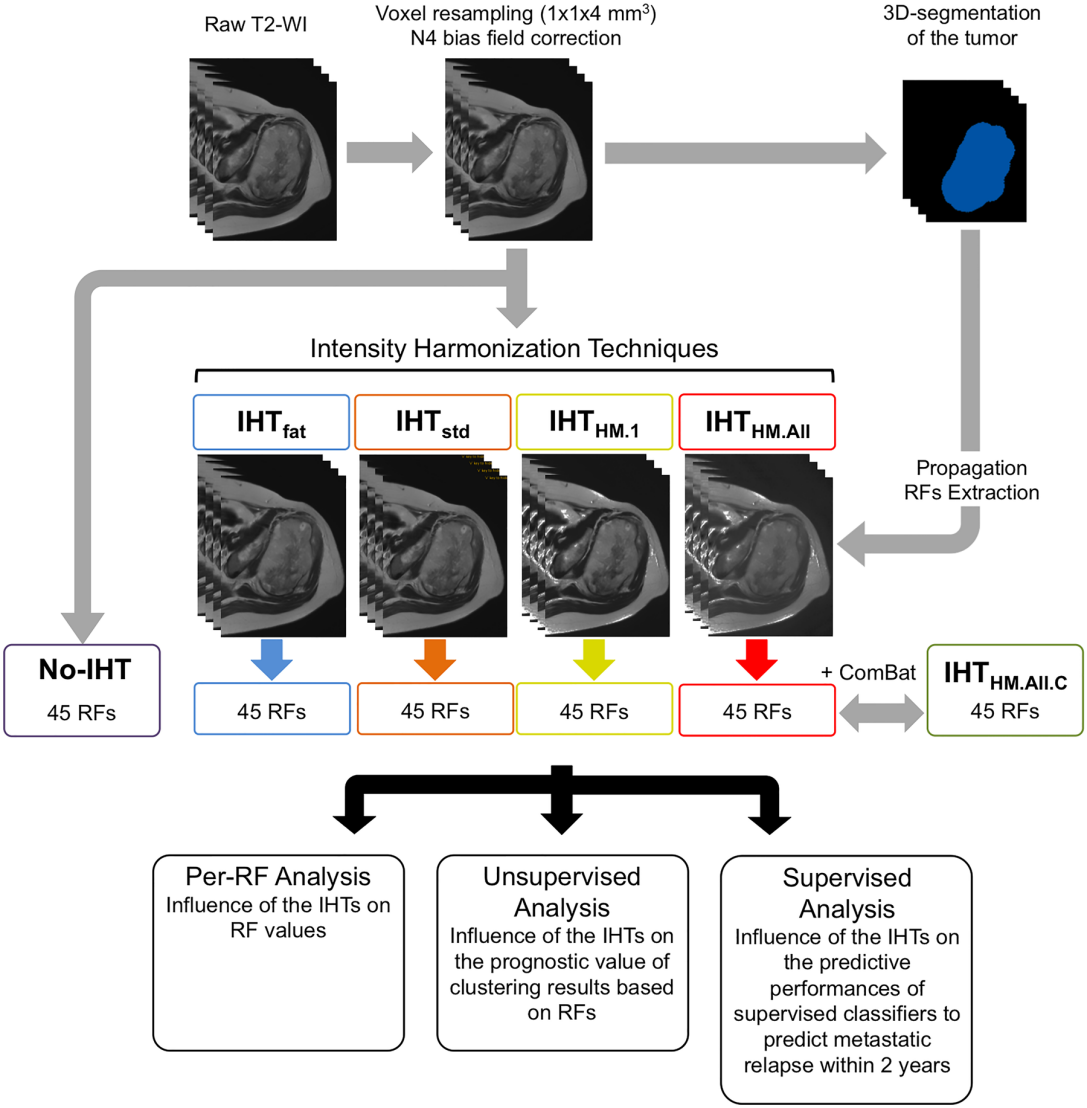
Study pipeline. *HM* histogram matching, *IHT* intensity harmonization technique, *No-IHT* no use of IHT before extracting radiomics features, *RF* radiomics features, *WI* weighted imaging.

After anonymizing MRIs, the postprocessing was performed with R (version 3.5.3, Vienna, Austria) by using the “oro.nifti”, “ANTsR” and “extranstr” packages^21^.

First, T2-WIs were converted to nifti format. Voxel size resampling (with b-spline interpolator) and N4 bias field correction were applied to obtain a common spatial resolution of 1 × 1 × 4 mm^3^ and to correct non-uniform intensities^22^.

Second, a senior radiologist (A.C., with 4 years of experience in sarcoma imaging) manually segmented the whole tumor volume, slice-by-slice, by using LIFEx freeware (version 5.10, Inserm, Orsay, France, www.lifexsoft.org)^23^. The radiologist had access to all the other MRI sequences to adjust the boundary of the segmentation if needed. The volumes of interests were all validated by a second senior radiologist (M.K., with 28 years of experience in sarcoma imaging).

Third, 4 IHTs were applied in parallel to the whole imaging dataset in order to harmonize the SIs of the T2-WI, providing 4 harmonized datasets, i.e.:

1. *IHT*_*fat*_*,* which consisted in dividing all the SIs of a given T2-WI by the mean SI of adipose tissue on that T2-WI, as follows:
  $${\mathrm{SI}\left(\mathrm{x},\mathrm{y},\mathrm{z}\right)}_{\mathrm{IHT}-\mathrm{fat}}=\frac{\mathrm{ SI}(\mathrm{x},\mathrm{y},\mathrm{z}) }{\mathrm{mean}(\mathrm{SI}(\mathrm{adipose tissue}))}$$
  where x, y and z are the coordinates of a voxel. To do so, the first senior radiologist segmented a volume of at least 10 cm^3^ of pure normally-appearing adipose tissue on each T2-WI in order to extract the mean SI per patient.
2. *IHT*_*std*_, which consisted in normalizing the SIs of a T2-WI according to the minimum and maximum of all voxels included in this T2-WI, as follows:
  $${\mathrm{SI}(\mathrm{x},\mathrm{y},\mathrm{z})}_{\mathrm{IHT}-\mathrm{std}}=\frac{\mathrm{SI}\left(\mathrm{x},\mathrm{y},\mathrm{z}\right)-\mathrm{min}(SIs)}{\mathrm{max}\left(SIs\right)-\mathrm{min}(SIs)}$$
3. *IHT*_*HM.1*_, which consisted in performing a matching of the intensity histogram of each T2-WI with the intensity histogram of a same normalized T2-WI from the same randomly chosen patient in the MRI dataset. This technique is achieved in 2 stages: first, a pre-specified number of percentiles and a reference image are given to the algorithm and, second, the new image is transformed according to several linear mapping of the SIs (depending on the number of landmarks) in order to match to the reference image (details about the conversion of SIs are given in Supplementary Data 1) (https://github.com/abdhigithub/hatch).
4. IHT_*HM.All*_, which consisted in performing a matching of the intensity histogram of each T2-WI with the average intensity histogram of the whole normalized MRI dataset.

*IHT*_*HM.All*_ and *IHT*_*HM.1*_ were trained on 100 histogram landmarks as a compromise between postprocessing time and image quality but other numbers of landmarks were tried (Supplementary Data 1). The superimposed SIs distributions of the 70 patients depending on the IHT are given in Supplementary Data 2.

### Radiomics features extraction

The tumor volumes were then propagated on the 4 post-processed imaging datasets (IHT_fat_, IHT_std_, IHT_HM.1_ and IHT_HM.All_) and on the imaging dataset without IHT (named No-IHT) enabling the extraction of 5 datasets of 45 3-D RFs by using LIFEx software. SIs were previously discretized into 128 fixed bins. Thirteen histogram-based and 32 s-order texture features from grey-level co-occurrence matrix (GLCM, n = 7—with a 1-voxel distance to neighbors), grey-level run length matrix (GLRLM, n = 11), neighborhood grey-level different matrix (NGLDM, n = 3) and grey-level zone length matrix (GLZLM, n = 11) were calculated (details are giving in Supplementary Data 3).

### ComBat compensation

We applied the ComBat-Harmonization function in R (https://github.com/fortin1/ComBatHarmonization) to the 45 RFs that were extracted from the IHT_HM.All_ dataset with a non-parametric setting in order to remove unwanted noise due to technical variations between the 3 MR-systems of the study while preserving biological variability, and notably when there are only a few patients per site^19,24,25^. ComBat-Harmonization is classically applied at the end of the postprocessing pipeline, herein, after the extraction of RFs obtained with the IHT that was hypothesized to be the more relevant and realistic among the 5 IHTs (namely IHT_HM.All_). This data-driven method identifies the protocol effect assuming that the value of each feature, RF, measured in a volume-of-interest, (x,y,z), with an imaging protocol, i, can be written as: RF_i,(x,y,z)_ = α + γ_i_ + δ_i_ × ε_i,(x,y,z)_ (in which α is the average value for features y_ij_; γ_i_ is an additive protocol effect and δ_i_ is a multiplicative protocol effect affected by an error term ε_ij_). The compensations consists in estimating the model parameters α, γ_i_ and δ_i_, and by using a maximum likelihood approach on the basis of the set of available observations:${RF}_{i,v(x,y,z)}^{ComBat}=\widehat{\alpha }+ \frac{{RF}_{i,v(x,y,z)}-\widehat{\alpha }-{\widehat{\gamma }}_{i} }{{\widehat{\delta }}_{i}}$, in which $\widehat{\alpha }$, ${\widehat{\gamma }}_{i}$ and ${\widehat{\delta }}_{i}$ are estimators of α, γ_i_ and δ_i_. Parametric and non-parametric forms of ComBat-Harmonization have been developed. The non-parametric form does not assume law followed by the parameters and has been used in the present study.

The resulting RFs were labelled IHT_HM.All.C_. In total, six paired datasets of 45 RFs were obtained, namely: No-IHT, IHT_fat_, IHT_std_, IHT_HM.1_, IHT_HM.All_ and IHT_HM.All.C_.

### Statistical analysis

Statistical analysis was performed with R. All tests were two-tailed. A p-value of less than 0.05 was deemed significant. A 3-steps approach was performed to evaluate the impact of IHTs on each aspect of radiomics studies (Fig. 1):

1. *Per-RF analysis*: RFs were all normalized in order to range between 0 and 1 and to facilitate direct comparisons. For each RF, the influence of the IHT was evaluated with one-way repeated-measures ANOVA. Post-Hoc comparisons were assessed with Tukey test and Bonferroni corrections. Intraclass correlation coefficients (ICC) were estimated for each RF, with a 2-way random model, agreement between raters and 6 raters (“irr” package).
2. *Unsupervised analysis:* A hierarchical clustering analysis with the Ward method was applied on each of the 6 subsets of RFs. RFs were centered and scaled by mean beforehand and the Euclidean distance between each pair of patients was computed. Visual inspection of the silhouette plot enabled to select 2 clusters of patients for each harmonization technique. We calculated the Baker’s gamma coefficient between each pair of dendrograms (dendextend” package), and the Kappa index between each pair of clustering results, which enabled the quantification of their divergence depending on the IHT^26^.
  The correlations between MFS and the clusters yielded by the models were assessed with Kaplan–Meier analysis and multivariable Cox models—after adjustment to the classical confounding covariables for sarcomas, i.e.: the longest baseline diameter (< vs. ≥ 10 cm), performance status (0 vs. 1–2), histological type (undifferentiated sarcomas vs. other), number of chemotherapy cycles (4 vs. 5–6), chemotherapy type (anthracycline-ifosfamide vs doxorubicine), adjuvant radiotherapy, surgical margins (R0 vs. R1-R2) and histological response (goods vs. poor responder to chemotherapy with a cut-off of 10% viable cells on post-chemotherapy surgical specimen). Prognostic performances of the 6 multivariate models were evaluated and compared through concordance-indices, which estimate the models’s ability to provide a reliable ranking of the survival times based on the individual risk scores.
3. *Supervised analysis*: The same supervised machine-learning approach was applied to the 6 datasets of RFs in order to predict the occurrence of a metastatic relapse within 2 years after curative surgery by using the “caret” and “glmnet” packages^27,28^. The total population of 70 patients with available clinical and radiological follow-up was randomly subdivided into one training cohort of 50 patients and one testing cohort of 20 patients with the same proportion of metastatic relapses by using the createDataPartition function. The training cohort was used to train a binomial logistic regression with combination of least absolute shrinkage and selection operator (LASSO) and ridge penalizations (elasticnet-LR). This algorithm consists of reducing the number and the importance of explanatory variables in order to optimize the performances of the classification model. The coefficients of the less contributive variables are shrunken towards 0 (: ridge regression) or even set to 0 (: LASSO). The amount of ridge and LASSO penalization was investigated by using a manual grid search with two hyperparamètres: α (mixing percentage) and λ (regularization parameter) and tenfold cross validation, repeated 5 times. The same partitioning of patients was used for the 6 datasets. The same clinical and pathological covariables as in the unsupervised analysis were included, in addition to the same 3 shape RFs (volume, compacity and sphericity—which are independent from the IHT).

The performances of supervised models were evaluated through cross-validated accuracy and area under the ROC curves (AUROC) with 95% confidence interval (95%CI). To do so, we extracted the 5 × 10 = 50 estimations of the accuracy and AUROC from the 50 distinct test sub-cohorts of 5 patients from the training cohort, and we applied the CI function from the Rmisc package to these vectors. Finally, for each RFs dataset, the final model with the highest AUROC in cross-validation was used on the testing cohort to estimate the AUROC and accuracy.

## Results

Thirty-two of the 70 patients (45.7%) were women with a median age of 58 (range: 19–84) **(**Table 1). The most frequent histological types were high-grade undifferentiated sarcomas (31/70, 44.3%), with a median size of 116 mm (range 40–273) and mostly deep-seated in the lower limb (35/70, 50%).

**Table 1:** Clinical and pathological features of the study population.

Characteristics	No. of patients
**Age (years old)**	
Median (range)	58 (19–84)
**Gender**	
Men	38/70 (54.3)
Women	32/70 (45.7)
**WHO performance status**	
PS 0	55/70 (78.6)
PS 1	15/70 (21.4)
**Histotype**	
Undifferentiated sarcoma	31/70 (44.3)
Synovial sarcoma	8/70 (11.4)
Rhabdomyosarcoma	8/70 (11.4)
Leiomyosarcoma	6/70 (8.6)
Myxoid/round cells liposarcoma	6/70 (8.6)
Pleomorphic sarcoma	3/70 (4.3)
Other sarcomas	8/70 (11.4)
**Longest diameter (mm)**	
median (range)	106 (40–273)
**Volume (cm**^**3**^**)**	
median (range)	220 (10.2–3,084)
**Location**	
Trunk	12/70 (17.1)
Shoulder girdle	9/70 (12.9)
Upper limb	9/70 (12.9)
Pelvic girdle	5/70 (7.1)
Lower limb	35/70 (50)
**Depth**	
Deep-seated	65/70 (92.9)
Superficial and aponeurotic	5/70 (7.1)
**No. of cycle**	
4 cycles	18/70 (25.7)
5–6 cycles	52/70 (74.3)
**Chemotherapy**	
Anthracycline-ifosfamide	64/70 (91.4)
Doxorubicine	6/70 (8.6)
**Adjuvant radiotherapy**	
No	5/70 (7.1)
Yes	65/70 (92.9)
**Margins**	
R0	41/70 (58.5)
R1	29/70 (41.4)
**Histological response**	
Good	16/70 (22.9)
Poor	54/70 (77.1)

Results are number of patients with percentage in parentheses, except for age, longest diameter and volume that are expressed as median with range in parentheses.

*WHO PS* World health organization performance status.

### Per-RF analysis

The influence of IHT was significant for all RFs (p-values range: < 0.0001–0.02, Supplementary Data 4). All significant differences in the RFs comparisons between each pair of post-processing techniques are listed in Table 2. The highest and lowest amounts of differences were obtained for post-hoc comparisons between IHT_HM-All_ and IHT_fat_ (31 statistically different RFs out of 45, 68.9%) and IHT_HM.All_ and IHT_HM.1_ (6/45, 13.3%), respectively.

**Table 2:** Summary of the per-radiomics features (RFs) analysis.

Post-hoc comparisons^a^	No. of significant differences^b^
IHT_HM.All_ vs IHT_fat_	31/45 (68.9%)
IHT_HM.All.C_ vs IHT_fat_	30/45 (66.7%)
IHT_HM.1_ vs IHT_fat_	30/45 (66.7%)
IHT_std_ vs IHT_HM.All_	28/45 (62.2%)
No-IHT vs IHT_fat_	28/45 (62.2%)
No-IHT vs IHT_HM.1_	28/45 (62.2%)
No-IHT vs IHT_HM.All_	27/45 (60%)
No-IHT vs IHT_HM.All.C_	27/45 (60%)
IHT_std_ vs IHT_HM.All.C_	27/45 (60%)
IHT_std_ vs No-IHT	23/45 (51.1%)
IHT_std_ vs IHT_fat_	20/45 (44.4%)
IHT_std_ vs IHT_HM.1_	19/45 (42.2%)
IHT_HM.1_ vs IHT_HM.All.C_	14/45 (31.1%)
IHT_HM.All.C_ vs IHT_HM.All_	13/45 (28.9%)
IHT_HM.1_ vs IHT_HM.All_	6/45 (13.3%)

^a^Post-Hoc comparisons correspond to the post-hoc Bonferroni-corrected Tukey tests for repeated-measures ANOVAs where the influence of the intensity harmonization techniques (IHT) on the 45 RFs was investigated.

^b^The number (no.) of significant differences corresponds to the number of RFs that were significantly different in a given post-hoc comparisons between 2 IHTs or the raw radiomics dataset, without IHT—named No-IHT (with percentage over the total number of RFs in parentheses).

*HM* histogram matching, *No.* number.

**Figure 2:**
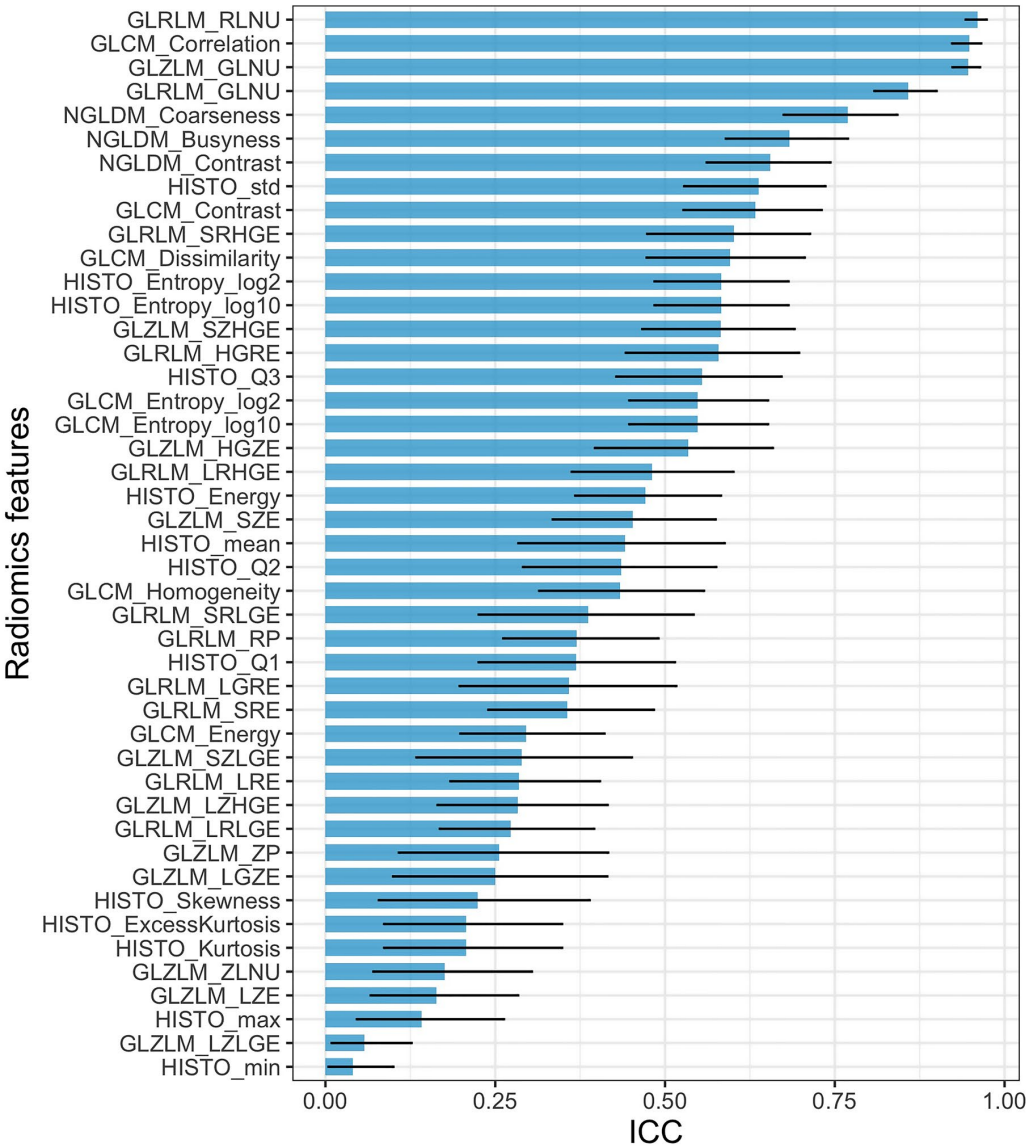
Intra-class correlation coefficients (ICC) of the radiomics features (RFs) depending on the intensity harmonization technique (IHT). Results are given with 95% confidence interval.

Figure 2 shows the 45 ICCs in descending order. The highest ICCs were reached with GLRLM_RLMNU, GLRLM_GLNU and GLCM_Correlation (≥ 0.95). The lowest ICCs were reached with GLZLM_ZLNU, GLZLM_LZE, HISTO_maximum, GLZLM_LZLGE and HISTO_minimum (< 0.20).

### Unsupervised analysis

**Figure 3:**
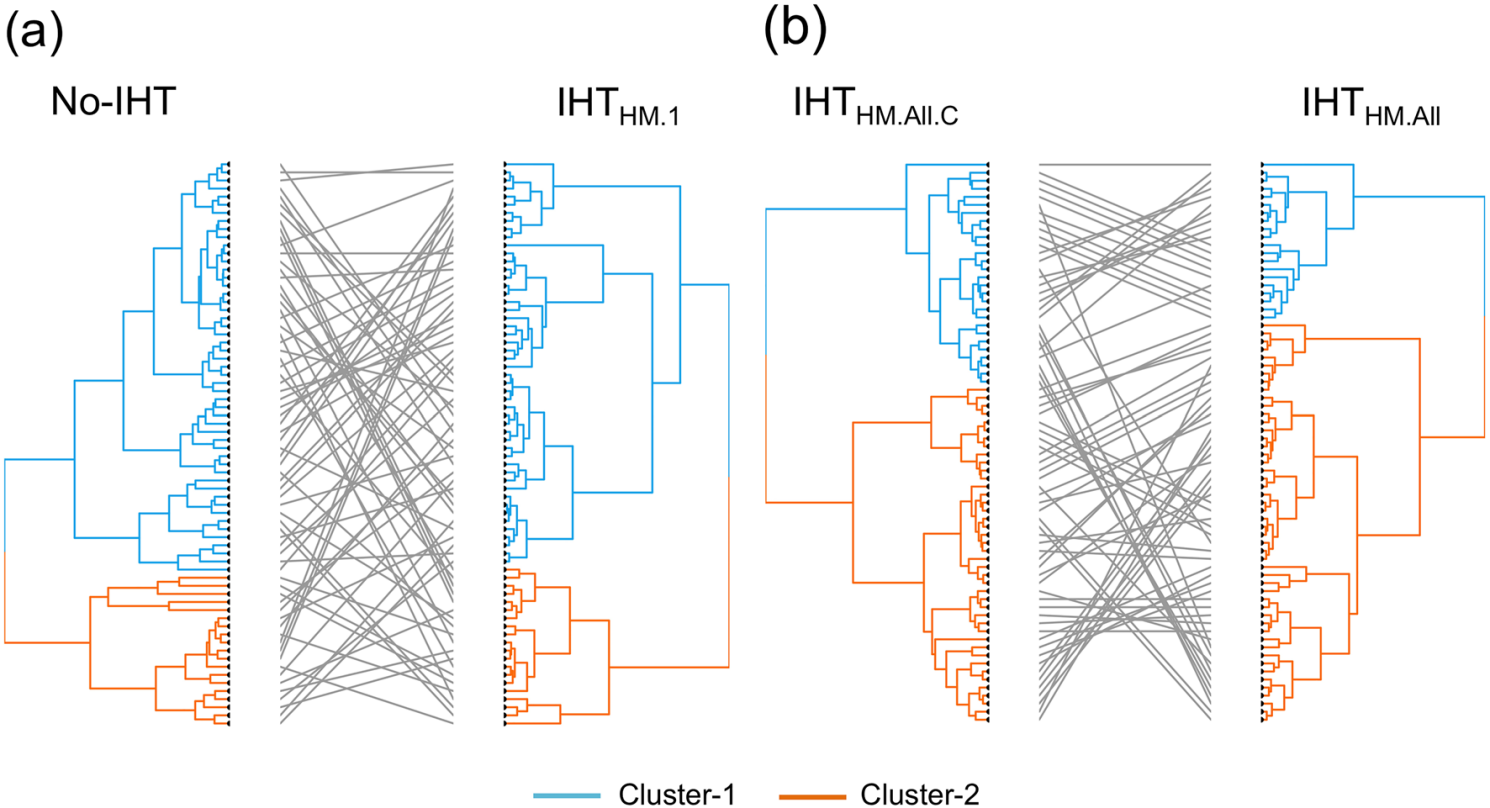
Comparisons of the hierarchical clustering results based on radiomics features from different datasets depending on the intensity harmonization technique (IHT) with: (**a**) the highest divergence, and (**b**) the lowest divergence. The dendrograms were obtained according to the following IHTs: histogram matching (HM) with a randomly-chosen normalized histogram of a patient (IHT_HM1_) versus no use of harmonization technique (No-IHT); and HM with the average normalized histogram of the study population (IHT_HM.All_) versus IHT_HM.All_ combined with ComBat harmonization method (IHT_HM.All.C_). By convention, cluster-1 (in blue) corresponds to the group of patients with the best prognosis regarding metastatic-relapse free survival.

All 6 unsupervised classifications achieved were different. Table 3 shows the correlation matrices for Kappa indices and Baker coefficients. The pair of clustering with the highest positive correlation was obtained with IHT_HM.All_ versus IHT_HM.All.C_ (Kappa = 0.75, Baker coefficient = 0.55). The lowest correlated pair was obtained with No-IHT versus IHT_HM.1_ (Kappa = 0.18, Baker coefficient = 0.05). Both correlated dendrograms are displayed in Fig. 3.

**Table 3:** Comparisons of the different dendrograms obtained by hierarchical clustering of the radiomics features with the 6 datasets depending on the intensity harmonization technique (IHT). (**a**) Corresponds to the Cohen’s Kappa index ranging from 0 (completely different clustering assignements) to 1 (exactly the same clustering assignements). (**b**) Corresponds to the the Baker’s gamma coefficient ranging from 0 (completely different dendrograms) to 1 (exactly the same two dendrograms).

(a)	IHT_fat_	IHT_std_	IHT_HM.1_	IHT_HM.All_	IHT_HM.All.C_	(b)	IHTfat	IHT_std_	IHT_HM.1_	IHT_HM.All_	IHT_HM.All.C_
No-IHT	0.40	0.33	0.18	0.39	0.35	No-IHT	0.19	0.11	0.05	0.05	0.07
	IHT_fat_	0.33	0.23	0.36	0.43		IHT_fat_	0.14	0.15	0.17	0.18
		IHT_std_	0.25	0.51	0.67			IHT_std_	0.11	0.30	0.42
			IHT_HM.1_	0.40	0.44				IHT_HM.1_	0.26	0.29
				IHT_HM.All_	0.75					IHT_HM.All_	0.55

**Figure 4:**
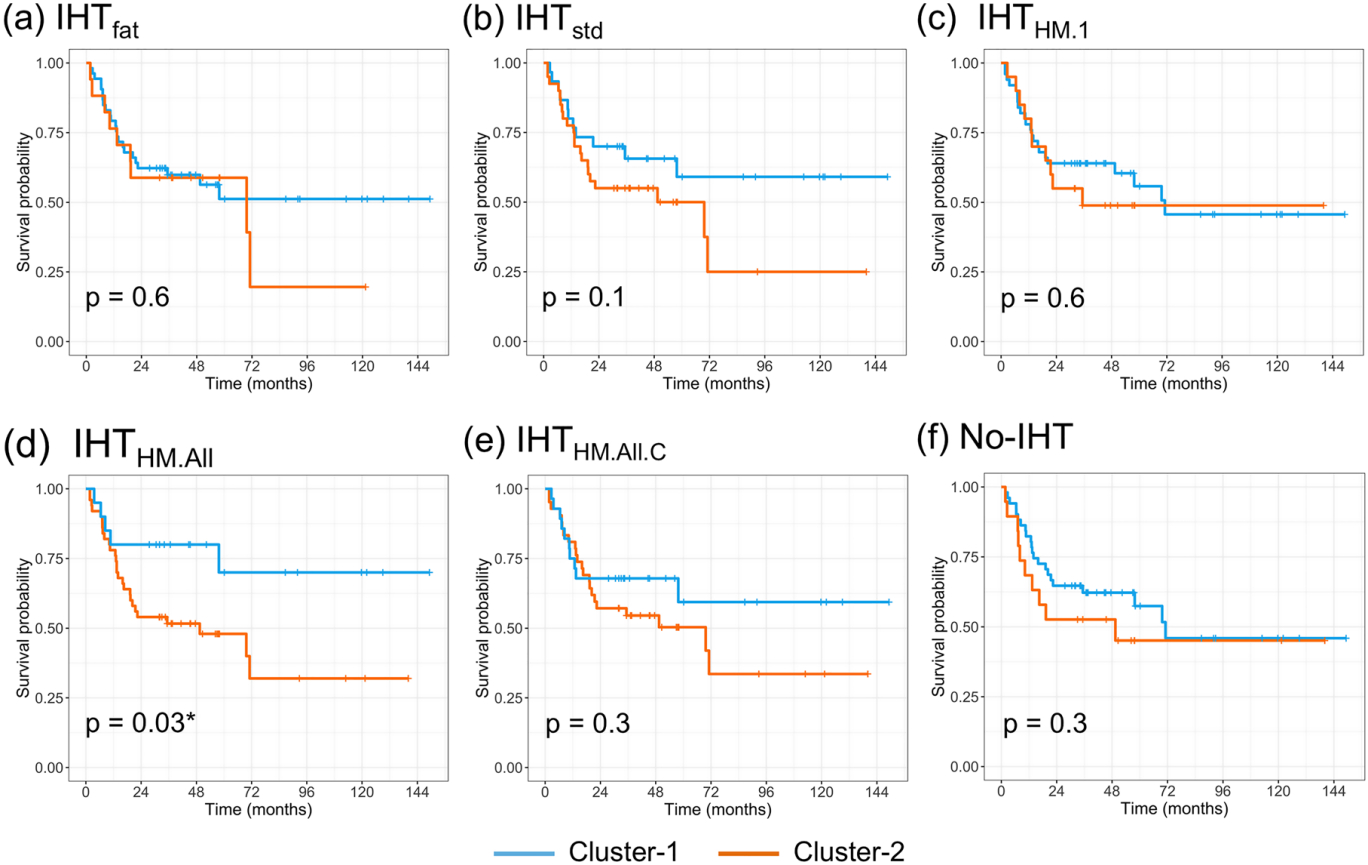
Kaplan–Meier curves for metastatic-relapse free survival depending on unsupervised clustering results based on radiomics features obtained with the different intensity harmonization techniques (IHT) or no use of harmonization technique (No-IHT).

Regarding the prognostic value of the clusters, our univariate analysis showed that significantly different survivals were found with the clusters obtained with the IHT_HM.All_ radiomics dataset (Log-rang p-value = 0.03) but not with the other IHTs. Kaplan Meier curves for the 6 clustering analyses are given in Fig. 4.

To assess the prognostic values in presence of confounding variables, we elaborated multivariate models demonstrating that the clusters obtained with RFs from the No-IHT, IHT_std_, IHT_HM.All_ and IHT_HM.All.C_ were independently associated with MFS in the multivariate modeling (p = 0.02, 0.007, 0.004 and 0.02, respectively—Table 4) but not the clusters obtained with RFs from the IHT_fat_ and IHT_HM.1_. Concordance-indices of the 6 prognostic models ranged from 0.71 (95% CI 0.67–0.75) for IHT_HM.1_ to 0.75 (95% CI 0.70–0.79) for No-IHT, IHTHM_std_ and IHT_HM.All_. The concordance-index of a reference prognostic model taking into account the clinical and pathological confounding co-variables alone was of 0.71 (95% CI 0.67–0.75).

**Table 4:** Unsupervised analysis based on radiomics features (RFs)—Prognostic value of the clustering results depending on the intensity harmonization technique (IHT).

Intensity harmonization technique	Clustering result	No. of patients	No. of events	2-years survival probability	Univariate analysis		Multivariate cox modeling^a^		
					Log-rank p-value	Concordance-index	HR	p-value	Concordance-index
No-IHT	Cluster-1	51	22	64.7 (52.8–79.3)	0.3	0.55 (0.50–0.59)	–	–	0.75 (0.71–0.79)
	Cluster-2	19	10	52.6 (34.4–80.6)			2.64 (1.15–6.04)	0.02*	
IHT_fat_	Cluster-1	53	23	62.3 (50.5–76.8)	0.6	0.51 (0.47–0.55)	–	–	0.72 (0.67–0.76)
	Cluster-2	17	9	58.8 (39.5–87.6)			1.65 (0.70–3.89)	0.3	
IHT_std_	Cluster-1	30	11	70 (55.4–88.5)	0.1	0.55 (0.50–0.60)	–	–	0.75 (0.72–0.79)
	Cluster-2	40	21	55 (41.6–72.8)			3.26 (1.48–7.71)	0.007*	
IHT_HM.1_	Cluster-1	50	22	64 (52–78.8)	0.6	0.52 (0.48–0.56)	–	–	0.71 (0.67–0.75)
	Cluster-2	20	10	55 (37–81.8)			1.52 (0.66–3.49)	0.3	
IHT_HM.All_	Cluster-1	20	5	80 (64.3–99.6)	0.03*	0.58 (0.54–0.62)	–	–	0.75 (0.70–0.79)
	Cluster-2	50	27	54 (41.8–69.7)			4.72 (1.64–13.56)	0.004**	
IHT_HM.All.C_	Cluster-1	28	10	67.9 (52.6–87.6)	0.3	0.53 (0.51–0.55)	–	–	0.73 (0.68–0.77)
	Cluster-2	42	22	57.1 (44–74.3)			2.89 (1.19–7.05)	0.02*	

Results for 2-years survival probability, hazard ratio and concordance-index are given with 95% confidence interval.

^a^Multivariate Cox modeling were adjusted for the following clinical and pathological covariables: performance status, histotype, initial longest diameter of the tumor, type of neoadjuvant chemotherapy, number of cycles of chemotherapy, surgical margins, histological response and adjuvant Radiotherapy.

*HM* histogram matching, *HR* hazard ratio, No: number.

*: p < 0.05, **: p < 0.005, ***: p < 0.001.

### Supervised analysis

In total, there were 29/70 (41.4%) metastatic relapses within the first two years of follow-up, which were distributed into 21/50 (42%) events in the training cohort and 8/20 (40%) events in the validation cohort.

The final hyperparameters and performances of the classification models are given in Table 5. The best performances in repeated cross-validation were found with the models based on the RFs from the IHTHM.All and IHTHM.1 datasets (AUROC = 0.71, 95% CI 0.66–0.76, and 0.69, 95% CI 0.64–0.74, respectively). The lowest AUROC was obtained with the No-IHT dataset (0.57, 95% CI 0.52–0.63).

**Table 5:** Accuracy and area under the ROC curves (AUROC) of the supervised models in repeated cross validation (training cohort) and in the testing/validation independent cohort, depending on the 5 intensity harmonization techniques (IHTs) or the lack of IHT (named No-IHT).

Intensity harmonization technique	Best hyperparameter tuning	Training cohort (results in repeated cross-validation)		Testing cohort	
		Accuracy	AUROC	Accuracy	AUROC
No-IHT	Alpha = 0.883 Lambda = 0.114	0.56 (0.52–0.64)	0.57 (0.52–0.60)	0.75 (0.51–0.89)	0.76 (0.50–1.0)
IHT_fat_	Alpha = 0.226, Lambda = 0.048	0.60 (0.64–0.55)	0.68 (0.63–0.73)	0.75 (0.51–0.91)	0.80 (0.56–1.0)
IHT_std_	Alpha = 0.384, Lambda = 0.086	0.63 (0.59–0.55)	0.64 (0.59–0.69)	0.70 (0.46–0.88)	0.69 (0.41–0.89)
IHT_HM.1_	Alpha = 0.394, Lambda = 0.200	0.62 (0.66–0.59)	0.69 (0.64–0.74)	0.75 (0.51–0.91)	0.82 (0.59–1)
IHT_HM.All_	Alpha = 0.338, Lambda = 0.384	0.61 (0.63–0.58)	0.71 (0.66–0.76)	0.60 (0.36–0.81)	0.77 (0.52–1)
IHT_HM.All.C_	Alpha = 0.166 Lambda = 0.840	0.58 (0.57–0.59)	0.68 (0.63–0.73)	0.60 (0.36–0.81)	0.71 (0.44–0.97)

Results are giving with 95% confidence interval.

**Figure 5:**
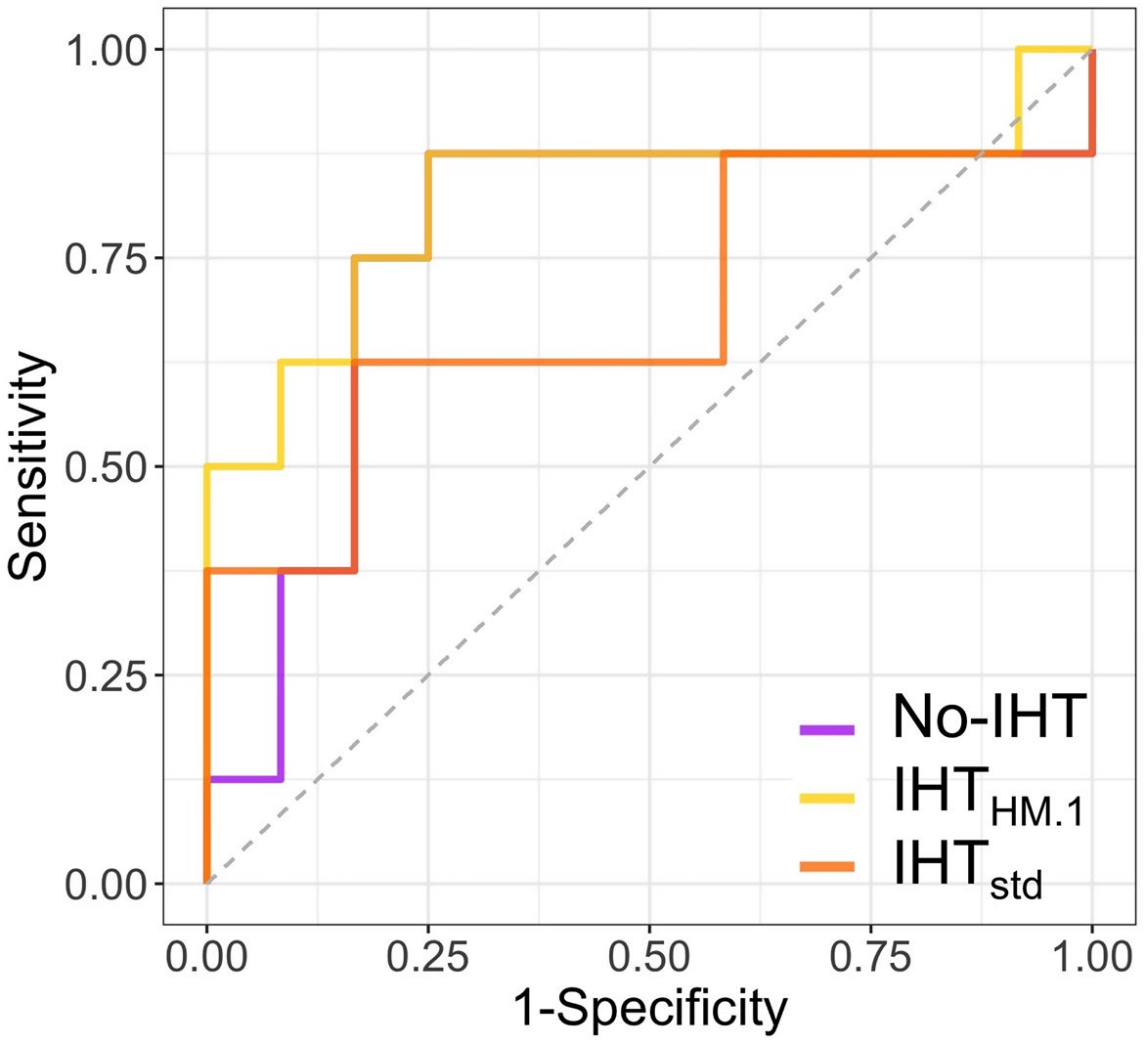
ROC curves for the best and worse supervised models to predict metastatic relapse within 2 years after the end of initial treatment in the testing cohort (built on the radiomics features from the IHT_HM.1_ and IHT_std_ datasets, respectively). The ROC curve of the final model without using harmonization technique (No-IHT) is also shown for comparison.

In descending orders, the AUROCs on the testing cohort were 0.82 (95% CI 0.59–1) with IHT_HM.1_, 0.80 (95% CI 0.56–1) with IHT_fat_, 0.77 (95% CI 0.52–1) with IHT_HM.All_, 0.76 (95% CI 0.50–01) with No-IHT, 0.71 (95% CI 0.444–0.973) with IHT_HM.All.C_, and 0.69 (95% CI 0.41–0.56) with IHT_std_. AUROCs of the most and less performant models and the No-IHT model in the testing cohort are shown in Fig. 5. The number of radiomics features included in the final models ranged from 3 (with No-IHT and IHTHM.AllC) to 21 (with IHTfat). Regarding the best final model, namely IHTHM.1, the number of selected radiomics features was of 7 out of 48 possible (by including the 3 shape features). Among these features, HISTO_Quartile1 and GLZLM_SZLGE were the most frequently selected (in 5 out of 6 models, and 4 out of 6 models, respectively) (Supplementary Data 5).

## Discussion

The post-processing of medical images to perform radiomics studies is mandatory to ensure the comparability of multicentric datasets but it can result in additional bias that may alter the performances of predictive models and preclude the reproducibility of MRI-based radiomics signatures. Because structural MRIs are acquired in arbitrary units, the intensity harmonization is crucial to enable the comparability of examinations acquired with different MR-systems, coils, and acquisition parameters. We found that all 45 textural features widely used in the literature were significantly influenced by IHT. Furthermore, depending on the IHC used, the results of unsupervised and supervised analyses based on RFs and their clinical correlations were dramatically changed. In addition, using an inappropriate IHT could decrease the performances of radiomics-based predictive models as it was highlighted by the comparative analysis with the models built with the No-IHT imaging dataset.

Our results concur with previous studies that found a significant influence of other post-processing steps on the absolute values of RFs (such as voxel size standardization, gray-levels discretization or manual segmentation) in addition to pre-processing steps (such as magnetic field strength, manufacturers, coils, acquisition parameters or filters). Recently, Scalco et al. found that the IHT for T2-WI had a significant impact on the reproducibility of RFs and on the inter-observer reproducibility of RFs that were extracted from pelvic organs from two MRIs separated by months^29^. These findings have been also applied to other IHTs such as variants of HM and a home-made method taking into account the SIs of organs of interest, the prostate, but the authors focused on the image, histogram and RFs values and not on RF-base predictions^30^. To our knowledge, this study is the first to demonstrate the dramatic impact of IHTs on RF-based predictions.

Moreover, in a recent review of MRI-based sarcoma radiomics studies, we found that 17 out 31 (54.8%) did not mention the method used for making comparable the SIs of MRI dataset (under review). It should be emphasized that the current Image Biomarker Standarisation Initiative and Radiomics Quality Score lack of precise guidelines regarding IHT for MRI^31^.

Previous studies have already emphasized the influence of IHT on segmentation and tissue classification tasks but they mostly involved brain MRI for inflammatory or degenerative diseases, and not specifically study their influence on radiomics analyses^24,25,32,33^. Moreover, the methods proposed in these studies were not readily transposable to non-brain imaging and/or not available in open source language (for instance, DeepHarmony)^34^.

In this study, we focused the analyses on techniques previously used in the body-imaging radiomics literature (i.e. scaling, histogram-matching or ComBat-Harmonization) but further studies should consider translating other popular intensity harmonization algorithms to body MRI. The RAVEL algorithm, which aims at estimating a voxel-specific unwanted variation by using a control region (i.e. brain cerebro-spinal fluid), may be particularly promising if applied to body-MR, with the possible use of healthy adipose tissues as control in the setting of soft tissue sarcomas for example^24,25^. Alternatively, instead of a post-processing intensity harmonization, the harmonization of SIs could be achieved since the acquisition step, through the use of standardized T1-mapping or T2-mapping sequence. However, thousands of MRIs have already been stored and, logically, the radiological community expects to pool and include these images in retrospective radiomics studies.

None of the IHTs used in this study demonstrated an unequivocal superiority compared to the others. This observation lets us hypothesize that the “best” technique is not universal but may actually vary depending on the dataset and the study objectives. Our present data does not allow us to validate this hypothesis, as it would require additional datasets to test if the same IHT constantly provides the best models whatever the disease and the outcome. While the unsupervised analysis highlighted the prognostic value of clusters elaborated with RFs from the IHT_std_, IHT_HM.All_ and IHT_HM.All.C_ datasets, the supervised analysis emphasized on the other hand the prognostic value of other models elaborated with RFs from the IHT_fat_ and IHT_HM.1_ in the testing cohort. It is worth noting that our supervised models showed moderately higher performances in the validation cohort than in the training cohort (range of differences: 0.03–0.13). Although this finding suggests that the models were not overfitted, it also indicates that the training could have been premature (despite the use of repeated cross-validation and exhaustive grid search) and that a sampling bias could have occurred during the data partitioning in our rather small study population (despite the fact that the splits were obtained randomly and were well-balanced regarding the outcome).

Importantly, our unsupervised analysis revealed that using an inappropriate IHT could even lead to a total loss of relevant information from the radiomics data. Indeed, the concordance indices of the reference model (which was elaborated with clinical and radiological variables alone) and the model relying on IHT_HM.1_ were equivalent, which stresses the lack of prognostic value of the corresponding clusters. Similarly, although the lowest AUROC was reached with the No-IHT dataset in cross-validation, the performances of this supervised model were not markedly different from those obtained with some of the IHTs in the two cohorts (especially the IHT_std_). These findings also suggest that radiomics studies should investigate all the available IHTs in an exploratory subset of the cohort, as well as no use of IHT, and subsequently select the one that optimizes the predictions. For instance, the extraction of RFs according to various voxel sizes and/or numbers of gray levels is commonly performed in radiomics studies. By analogy, one could consider extracting the RFs according to different IHTs and select the most robust and predictive RFs at univariable level. Hence, the intensity harmonization techniques could be considered as a “hyperparameter” of the post-processing pipeline. Interestingly, IHT_HM.All.C_ yielded moderately good performances in both unsupervised and supervised analyses (with similar results in training and testing cohorts), which suggests that this method may provide the more realistic radiomics data in the setting of our study. It should be emphasized that the co-variable arguments given to the ComBat function may/might be incomplete in the setting of sarcomas. In any case, the clinical outcome of the study should not be included among the ComBat covariables because it should not depend on the MR-system or acquisition parameters of the sequences. A distinctive feature of sarcomas over other cancers is their anatomical ubiquity, hence, requiring adjusting several other acquisition parameters depending on the tumor location (for instance thoracic wall, thigh or wrist). Further studies should investigate the best co-variables for ComBat for non-brain MRI. In addition, ComBat could have been used with the No-IHT, IHT_fat_, IHT_std_, IHT_HM.1_ radiomics features. We purposely decided to limit the application of ComBat to only one dataset (IHT_HM.All_) to avoid multiplying the post-hoc analyses, performances measurements, or superposing ROC curves, while our current results already enables us to stress the strong impact of IHT on radiomics-features and radiomics-based classifications and predicitions.

Our results also deepened that intra-tumoral heterogeneous SIs on T2-WI is predictive of MFS in a quantitative manner and other studies have also correlated this parameter with overall and/or metastatic-relapse free survivals in STS patients with relatively close and similar performances to ours^6,7,20^. Indeed, Peeken et al. used an equivalent of IHTstd and applied ComBat to correct for multicenter effect. They also provided the sarcoma histological type as a biological covariable (which slightly improved the performances)^6^. Their best model relied on radiomics features from Fat Sat T2 weighted imaging and showed a concordance-index of 0.74 in the validation cohort. On the other hand, Spraker et al. did not explicitly use an intensity harmonization technique, neither ComBat^7^. Interestingly, their best clinical and radiological prognostic models for the overall survival showed a concordance-index of 0.78 in the validation cohort.

Our study has limits. First, the study population was relatively small although this is the largest study investigating IHT and radiomics. It should be noted sarcoma radiological studies rarely exceed our population number. Second, we focused this proof-of-concept methodological study on T2-WI sequences but further investigations should be performed on other MRI sequences, such as T1-WI, contrast-enhanced T1-WI, DCE-MRI and diffusion imaging. We purposely chose this sequence because it is commonly reported as the most informative morphological sequence for sarcomas^8,20^. Third, our study design could be criticized. Indeed, judging which of the IHTs is the best by using the performances of predictive models (AUROC or concordance-index) as judgment criteria can only be valid if the intrinsic prognostic value of MRI-based radiomics features is certain. In this case, lowering these performances with a particular IHT would mean that this IHT caused noise and inappropriate deviation in the data. However, as already stated, prior studies converged towards same results regarding the relationship between MRI-based radiomics features, heterogeneity on T2-WI and outcomes of sarcoma patients^6,7,20,35^. Alternative study designs could have been proposed in the absence of such relationship, (i) either by using a phantom made of compartments with various degrees of heterogeneity, (ii) or by using MRIs of healthy volunteers covering organs with different textures and investigating which IHT enables the best radiomics-based classification of these organs (by analogy with the study by Orlhac et al.)^19^. Fourth, other shape and textural RFs than the 48 features used in this study can be encountered in the literature. Yet, we purposely decided to limit our investigations to this set of RFs, which are proposed by the LIFEx freeware, as they follow the definitions of the Imaging Biomarker Standardization Initiative^23,31^. Furthermore, adding more potential radiomics predictors in our multivariate analyses would have increased the multidimensionality of our dataset and the risk of overfitted results regarding the limited number of patients.

To conclude, through the example of sarcomas, our study highlights that the IHT can directly influence the values of MRI-based RFs, subsequently leading to dramatical changes in the predictions of both unsupervised and supervised models. Therefore, IHTs need to be deepened regarding non-brain MRI and should be carefully explored and detailed when building radiomics models to ensure the robustness and reproducibility of radiomics signatures.

## Supplementary information

Supplementary file1

## Data Availability

The datasets generated during and/or analyzed during the current study are not publicly available due to the clinical and confidential nature of the material but can be made available from the corresponding author on reasonable request.
