# Supplementary material for: Intensity harmonization techniques influence radiomics features and radiomics-based predictions in sarcoma patients
Source: Sci Rep. 2020 Sep 23;10:15496. doi: 10.1038/s41598-020-72535-0 (PMC7511974; doi:10.1038/s41598-020-72535-0)
Supplement: Supplementary file 1 — Supplementary file1 [file 41598_2020_72535_MOESM1_ESM.docx]

**SUPPLEMENTARY DATA**

**Supplementary Data 1.** Principle of histogram matching (HM) of MRIs with a standard reference.

The method was developed by Nyul and Udupa (1999, 2000) and consists in two steps.

Let consider:

- p_1_: the signal intensity (SI) of the minimum percentile

- p_2_: the SI of the maximum percentile

- N: be the N-th percentile

- i: the i-th value of percentiles

- μ_Ni_: the SI of the Ni-th percentile

- s_1_: the minimum SI of the stardized image of interest

- s_2_: the minimum SI of the stardized image of interest

- the landmarks L = {p_1_; μ_N1_; μ_N2_; …; μ_Ni_; …; p_2_}

The first step is a training transformation in which the histogram H_j_ of an image j is calculated. The SI value p_1,j_ and p_2,j_ corresponding to p1 and p2 and the landmarks location μ_Ni_ on Hj are determined (**a**). Of note, the formula to transform x ∈ [p_1,j_ ; p_2,j_] in x’ ∈ [s_1_ ; s_2_] is given as follows: $x^{'}=s_{1}+\frac{x-p_{1,j}}{p_{2,j}-p_{1,j}} \times(s_{2}-s_{1})$

The next step is the transformation step. Several linear mapping are performed to transform each segment [μ_Ni,j_ ; μ_Ni+1,j_] into [μ_Ni_ ; μ_Ni+1_] (**b**).


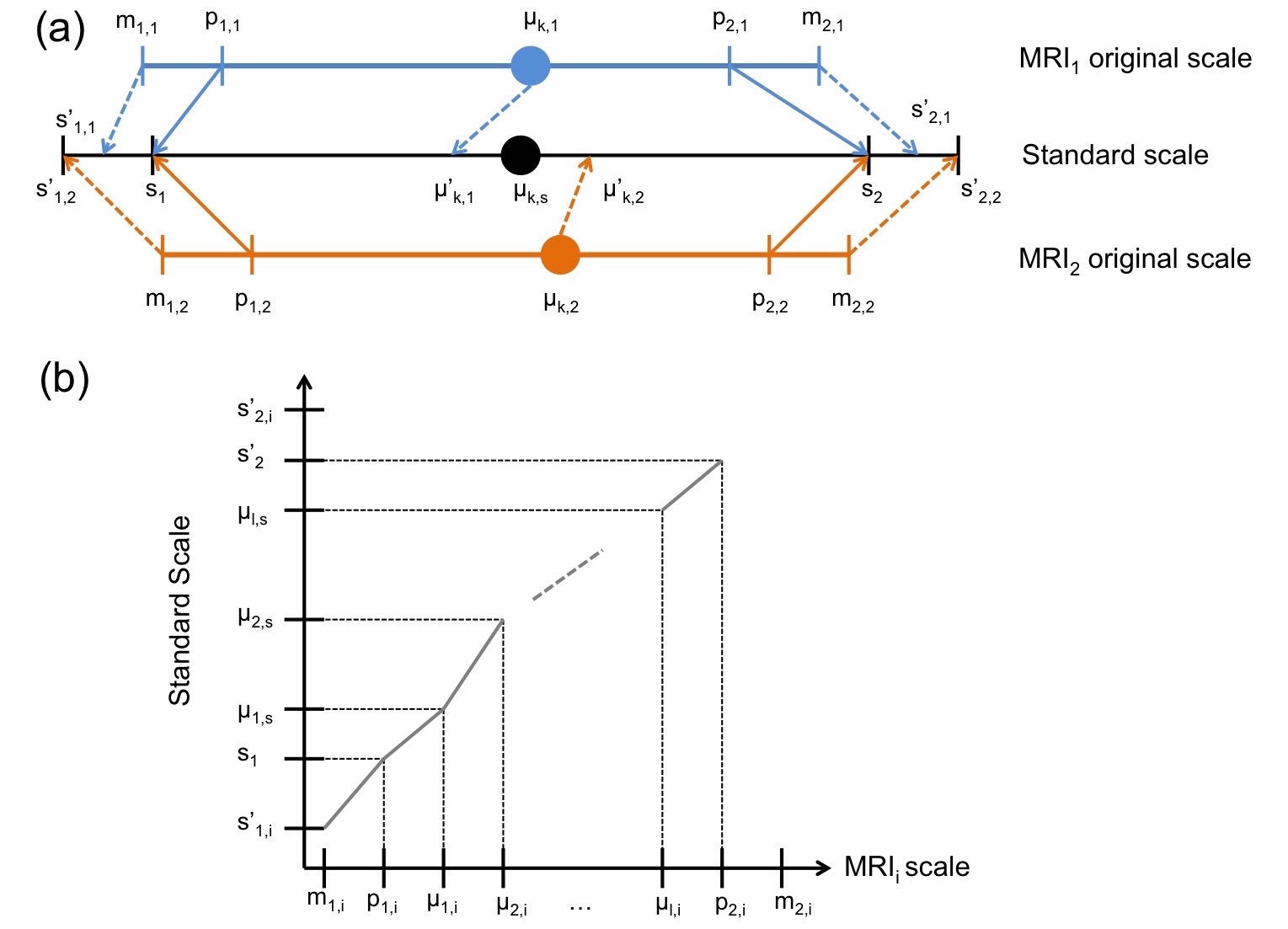


It should be noted that the predefined number of landmarks influences the visual aspect (**c**) and the histogram of signal intensities (SIs) of images (**d**), here of an axial T2-weighted imaging of a patients with an undifferentiated pleomorphic sarcoma in the left iliac muscle (lm: landmark).


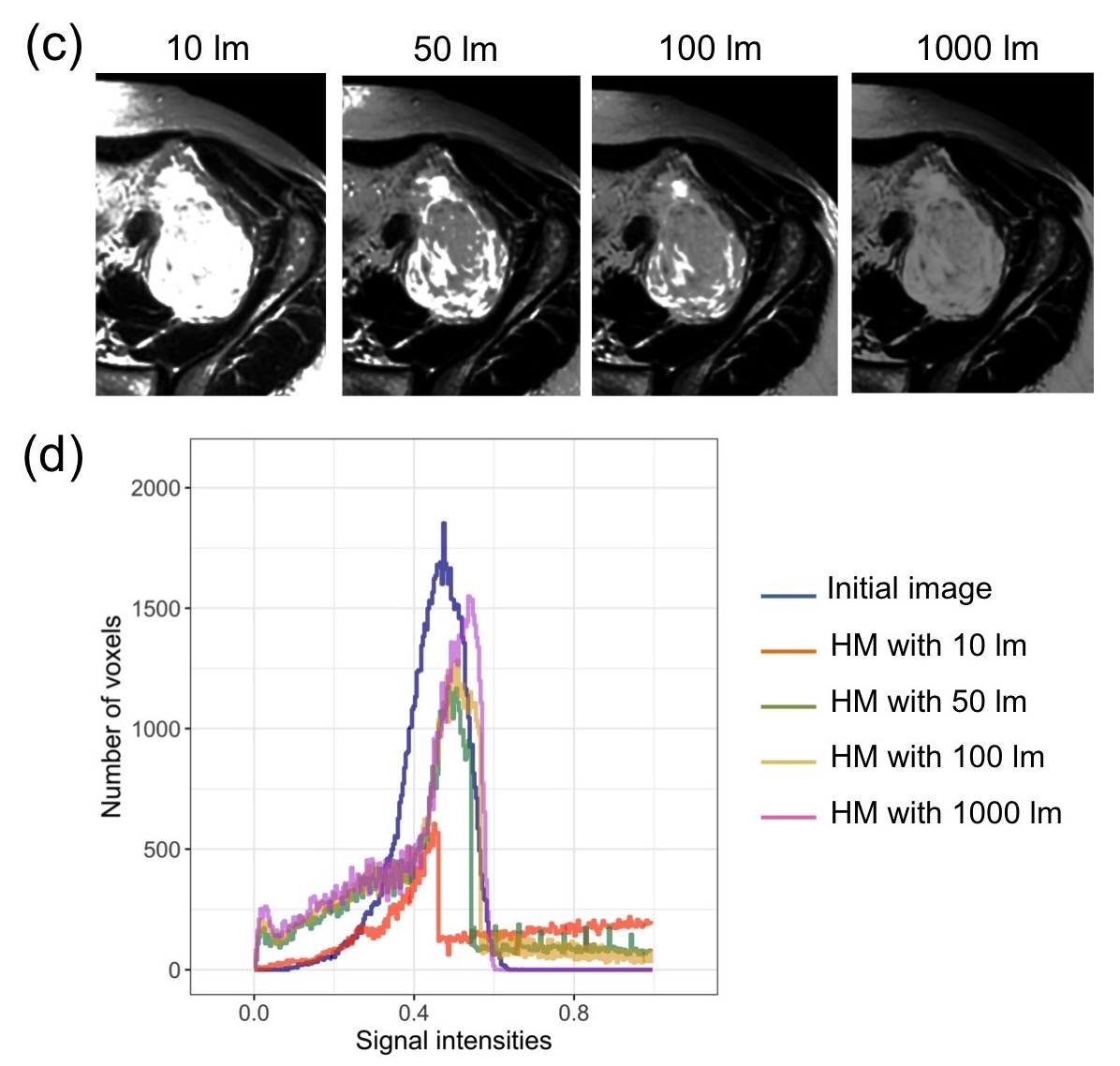


**Supplementary Data 2.** Distribution of the signal intensities in the whole population before applying intensity harmonization techniques (IHTs), which was named No-IHT (**a**), and after applying IHT_fat_ (**b**), IHT_std_ (**c**), IHT_HM.1_ (**d**) and IHT_HM.All_ (**e**). Each line represents the histogram of one patient. Raw T2-weighted imagings (T2-WI) were voxel-size resampled and N4 bias-field corrected beforehand.


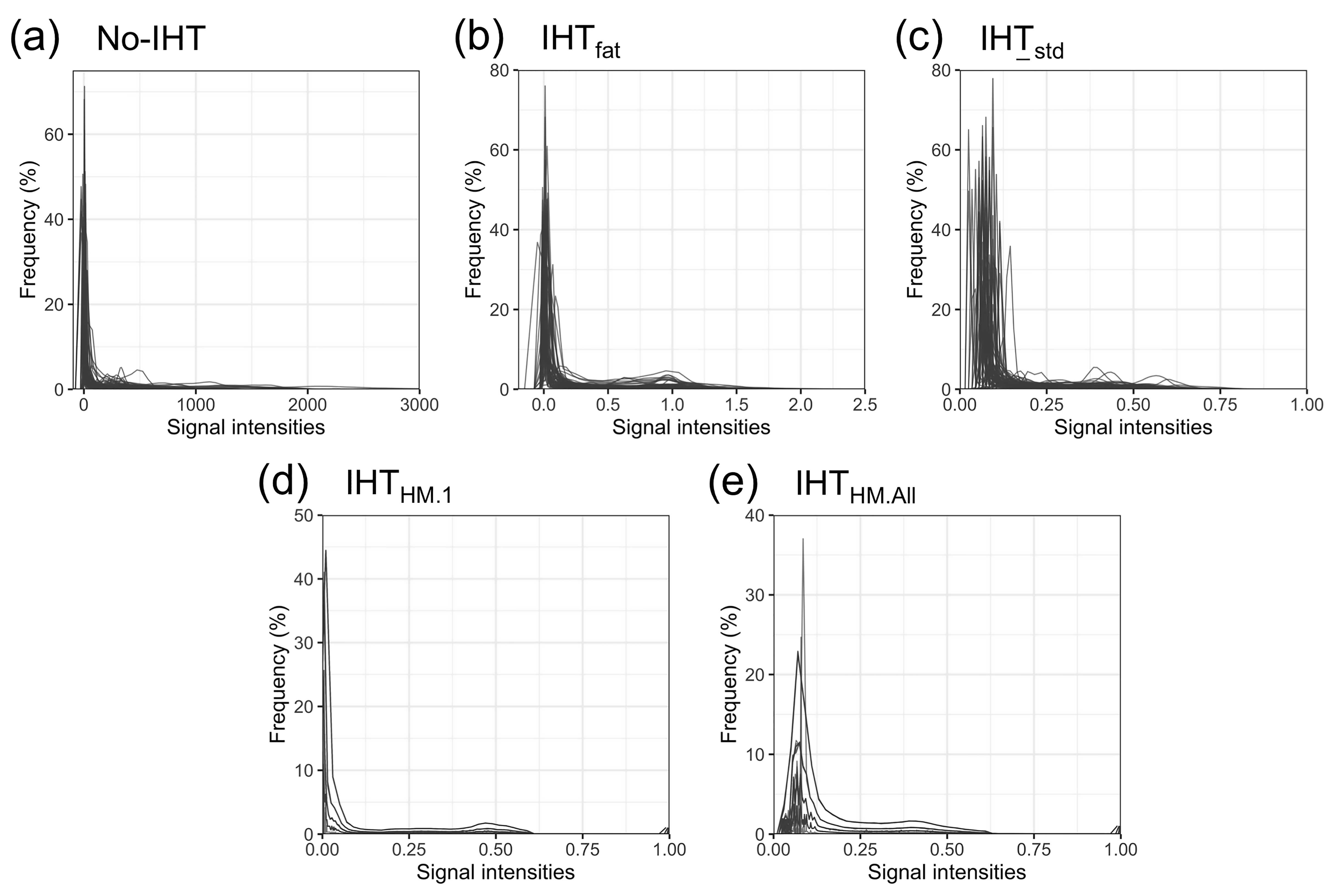


**Supplementary Data 3.** Textural radiomics features (RFs) used in the study. RFs were extracted on 3D tumor volume with LIFEx freeware (Inserm, Orsay, France). Details regarding the formula can be found at: [https://www.lifexsoft.org/index.php/resources/19-texture/radiomic-features?filter_tag[0]=](https://www.lifexsoft.org/index.php/resources/19-texture/radiomic-features?filter_tag%5B0%5D=)

| **First-order texture features** | | **Grey-level co-occurrence matrix features (GLCM)** |
| --- | --- | --- |
| HISTO_min | HISTO_Quartile_1 | GLCM_Homogeneity |
| HISTO_mean | HISTO_Quartile_2 | GLCM_Energy |
| HISTO_std | HISTO_Quartile_3 | GLCM_Contrast |
| HISTO_max |  | GLCM_Correlation |
| HISTO_Skewness |  | GLCM_Entropy_log10 |
| HISTO_Kurtosis |  | GLCM_Entropy_log2 |
| HISTO_ExcessKurtosis |  | GLCM_Dissimilarity |
| HISTO_Entropy_log10 |  |  |
| HISTO_Entropy_log2 |  |  |
| HISTO_Energy |  |  |
|  |  |  |
| **Grey-level run length matrix features(GLRLM)** | **Neighborhood grey-level different matrix features (NGLDM)** | **Grey-level zone length matrix features (GLZLM)** |
| GLRLM_SRE | NGLDM_Coarseness | GLZLM_SZE |
| GLRLM_LRE | NGLDM_Contrast | GLZLM_LZE |
| GLRLM_LGRE | NGLDM_Busyness | GLZLM_LGZE |
| GLRLM_HGRE |  | GLZLM_HGZE |
| GLRLM_SRLGE |  | GLZLM_SZLGE |
| GLRLM_SRHGE |  | GLZLM_SZHGE |
| GLRLM_LRLGE |  | GLZLM_LZLGE |
| GLRLM_LRHGE |  | GLZLM_LZHGE |
| GLRLM_GLNU |  | GLZLM_GLNU |
| GLRLM_RLNU |  | GLZLM_ZLNU |
| GLRLM_RP |  | GLZLM_ZP |

**Supplementary Data 4.** Detailed results of the one-way repeated-measures ANOVAs

| **Radiomics Features** | **F-value** | **P-value** | **P-values of the post-hoc comparisons** | | | | | | | | | | | | | | |
| --- | --- | --- | --- | --- | --- | --- | --- | --- | --- | --- | --- | --- | --- | --- | --- | --- | --- |
|  |  |  | **IHT_HM.All_ vs.**  **IHT_fat_** | **IHT_HM.AllC_ vs.**  **IHT_fat_** | **IHT_HM.1_ vs. IHT_fat_** | **No-IHT vs. IHT_fat_** | **IHT_std_ vs. IHT_fat_** | **IHT_HM.AllC_**  **vs.**  **IHT_HM.All_** | **IHT_HM.1_ vs. IHT_HM.All_** | **No-IHT vs. IHT_HM.All_** | **IHT_std_**  **vs. IHT_HM.All_** | **IHT_HM.1_**  **vs. IHT_HM.AllC_** | **No-IHT**  **vs.**  **IHT_HM.AllC_** | **IHT_std_**  **vs. IHT_HM.AllC_** | **No-IHT vs. IHT_HM.1_** | **IHTstd vs. IHT_HM.1_** | **IHTstd vs.**  **No-IHT** |
| HISTO_min | 940.3 | <0.0001 | <0.0001 | <0.0001 | <0.0001 | <0.0001 | <0.0001 | 0.0351 | 1 | <0.0001 | <0.0001 | 0.0564 | <0.0001 | <0.0001 | <0.0001 | <0.0001 | <0.0001 |
| HISTO_mean | 44.5 | <0.0001 | <0.0001 | <0.0001 | <0.0001 | <0.0001 | 1 | 1 | 1 | <0.0001 | <0.0001 | 0.7947 | <0.0001 | 0.0002 | <0.0001 | <0.0001 | <0.0001 |
| HISTO_std | 16.7 | <0.0001 | 0.1593 | 0.0442 | 0.0771 | 1,00E-04 | 1 | 1 | 1 | <0.0001 | 1 | 1 | <0.0001 | 1 | <0.0001 | 1 | <0.0001 |
| HISTO_max | 108.8 | <0.0001 | <0.0001 | 1 | <0.0001 | 1 | <0.0001 | 0.0111 | 1 | <0.0001 | <0.0001 | 0.003 | 0.7804 | <0.0001 | <0.0001 | <0.0001 | <0.0001 |
| HISTO_Q1 | 43.2 | <0.0001 | <0.0001 | <0.0001 | <0.0001 | 0.003 | 1 | 1 | 1 | <0.0001 | <0.0001 | 1 | <0.0001 | <0.0001 | <0.0001 | <0.0001 | 0.0006 |
| HISTO_Q2 | 36.4 | <0.0001 | <0.0001 | <0.0001 | <0.0001 | <0.0001 | 1 | 1 | 1 | <0.0001 | 0.0003 | 1 | <0.0001 | 0.0001 | <0.0001 | <0.0001 | <0.0001 |
| HISTO_Q3 | 22.9 | <0.0001 | 1 | 1 | 0.0171 | <0.0001 | 1 | 1 | 0.4553 | <0.0001 | 1 | 0.1259 | <0.0001 | 1 | <0.0001 | 0.81 | <0.0001 |
| HISTO_Skewness | 138.8 | <0.0001 | <0.0001 | <0.0001 | <0.0001 | 1 | 1 | 0.6287 | 1 | <0.0001 | <0.0001 | 1 | <0.0001 | <0.0001 | <0.0001 | <0.0001 | 1 |
| HISTO_Kurtosis | 82.6 | <0.0001 | <0.0001 | <0.0001 | <0.0001 | 1 | 1 | 0.2638 | 1 | <0.0001 | <0.0001 | 0.2628 | <0.0001 | <0.0001 | <0.0001 | <0.0001 | 1 |
| HISTO_ExcessKurtosis | 82.6 | <0.0001 | <0.0001 | <0.0001 | <0.0001 | 1 | 1 | 0.2638 | 1 | <0.0001 | <0.0001 | 0.2628 | <0.0001 | <0.0001 | <0.0001 | <0.0001 | 1 |
| HISTO_Entropy_log10 | 3.4 | 0.005 | 0.0028 | 0.0677 | 0.0151 | 0.2053 | 0.3797 | 1 | 1 | 1 | 1 | 1 | 1 | 1 | 1 | 1 | 1 |
| HISTO_Entropy_log2 | 3.4 | 0.005 | 0.0028 | 0.0677 | 0.0151 | 0.2053 | 0.3797 | 1 | 1 | 1 | 1 | 1 | 1 | 1 | 1 | 1 | 1 |
| HISTO_Energy | 4.5 | 0.0005 | 0.0995 | 1 | 0.0245 | 1 | 0.0461 | 1 | 1 | 0.0516 | 1 | 1 | 1 | 1 | 0.0117 | 1 | 0.0228 |
| GLCM_Homogeneity | 19.6 | <0.0001 | <0.0001 | <0.0001 | <0.0001 | <0.0001 | <0.0001 | 0.4766 | 1 | 1 | 1 | 0.8613 | 1 | 0.6437 | 1 | 1 | 1 |
| GLCM_Energy | 9.2 | <0.0001 | 0.2225 | 1 | 0.0183 | 0.4364 | 0.0001 | 0.0035 | 1 | 1 | 0.4588 | 0.0001 | 0.0092 | <0.0001 | 1 | 1 | 0.235 |
| GLCM_Contrast | 14.5 | <0.0001 | 0.0002 | 1 | 0.2479 | 0.0028 | 1 | 0.0172 | 0.8227 | <0.0001 | 0.0016 | 1 | <0.0001 | 1 | <0.0001 | 0.7478 | 0.0005 |
| GLCM_Correlation | 15.4 | <0.0001 | <0.0001 | 0.2338 | 1 | 1 | 1 | 0.0012 | <0.0001 | <0.0001 | <0.0001 | 1 | 0.0063 | 0.0267 | 0.1358 | 0.4114 | 1 |
| GLCM_Entropy_log10 | 3.9 | 0.002 | 0.003 | 0.1598 | 0.0081 | 0.0064 | 0.038 | 1 | 1 | 1 | 1 | 1 | 1 | 1 | 1 | 1 | 1 |
| GLCM_Entropy_log2 | 3.9 | 0.002 | 0.003 | 0.1598 | 0.0081 | 0.0064 | 0.038 | 1 | 1 | 1 | 1 | 1 | 1 | 1 | 1 | 1 | 1 |
| GLCM_Dissimilarity | 21.9 | <0.0001 | <0.0001 | 2,00E-04 | 0.0017 | 0.0951 | 1 | 0.8659 | 0.2844 | <0.0001 | <0.0001 | 1 | <0.0001 | 0.0033 | <0.0001 | 0.0174 | 0.0124 |
| GLRLM_SRE | 23.3 | <0.0001 | <0.0001 | <0.0001 | <0.0001 | <0.0001 | <0.0001 | 0.2766 | 1 | 1 | 0.1519 | 0.0132 | 0.2193 | <0.0001 | 1 | 1 | 0.1934 |
| GLRLM_LRE | 18 | <0.0001 | 0.0947 | <0.0001 | 1 | 1 | 0.3328 | 0.0026 | 0.1378 | 1 | <0.0001 | <0.0001 | <0.0001 | <0.0001 | 1 | 0.2371 | 0.0007 |
| GLRLM_LGRE | 61.2 | <0.0001 | <0.0001 | <0.0001 | <0.0001 | <0.0001 | <0.0001 | 0.4426 | 1 | 0.0038 | 0.0049 | 1 | <0.0001 | 1 | <0.0001 | 0.7949 | <0.0001 |
| GLRLM_HGRE | 28.5 | <0.0001 | <0.0001 | <0.0001 | <0.0001 | <0.0001 | 1 | 1 | 0.7298 | 0.0637 | <0.0001 | 0.8421 | 0.0772 | <0.0001 | 1 | <0.0001 | <0.0001 |
| GLRLM_SRLGE | 55.1 | <0.0001 | <0.0001 | <0.0001 | <0.0001 | <0.0001 | <0.0001 | 0.3531 | 0.0272 | 0.001 | 0.0892 | 1 | <0.0001 | 1 | <0.0001 | 1 | <0.0001 |
| GLRLM_SRHGE | 24.9 | <0.0001 | 0.0001 | 0.0001 | <0.0001 | <0.0001 | 1 | 1 | 0.7742 | 0.0008 | <0.0001 | 0.9326 | 0.0011 | <0.0001 | 0.5244 | <0.0001 | <0.0001 |
| GLRLM_LRLGE | 24.7 | <0.0001 | <0.0001 | 0.0002 | <0.0001 | <0.0001 | <0.0001 | 0.56 | 0.4539 | 0.0604 | 1 | 0.0003 | <0.0001 | 0.0016 | 1 | 1 | 1 |
| GLRLM_LRHGE | 18.1 | <0.0001 | 1 | <0.0001 | 1 | <0.0001 | 0.0002 | <0.0001 | 1 | <0.0001 | 0.0011 | <0.0001 | 1 | 1 | <0.0001 | 0.0245 | 0.1587 |
| GLRLM_GLNU | 2.7 | 0.0224 | 1 | 1 | 1 | 1 | 1 | 0.75 | 1 | 1 | 1 | 0.0096 | 0.2484 | 0.1493 | 1 | 1 | 1 |
| GLRLM_RLNU | 11.9 | <0.0001 | 0.8615 | 0.0013 | 0.3026 | 1 | 0.5392 | <0.0001 | 1 | 0.0048 | 0.001 | <0.0001 | 0.3941 | 1 | 0.0009 | 1,00E-04 | 1 |
| GLRLM_RP | 14.8 | <0.0001 | 0.0104 | 0.9744 | 4,00E-04 | <0.0001 | <0.0001 | 1 | 1 | 0.3628 | 0.0003 | 0.298 | 0.0022 | <0.0001 | 1 | 0.0072 | 0.6532 |
| NGLDM_Coarseness | 21.2 | <0.0001 | 0.5523 | 1 | 0.3841 | <0.0001 | 1 | 1 | 1 | <0.0001 | 1 | 1 | <0.0001 | 1 | <0.0001 | 0.8485 | <0.0001 |
| NGLDM_Contrast | 7 | <0.0001 | 0.3646 | 0.5379 | 1 | 0.0525 | 1 | 0.0002 | 1 | <0.0001 | 0.0349 | 0.1225 | 1 | 1 | 0.0079 | 1 | 0.5009 |
| NGLDM_Busyness | 10.9 | <0.0001 | 1 | 1 | 0.064 | 1 | <0.0001 | 1 | 0.53 | 0.1862 | 0.001 | 0.0357 | 1 | <0.0001 | 0.0001 | 0.9112 | <0.0001 |
| GLZLM_SZE | 18.4 | <0.0001 | 1 | 0.002 | 1 | <0.0001 | 1 | 0.6665 | 1 | <0.0001 | 0.0091 | 0.6106 | 0.018 | <0.0001 | <0.0001 | 0.0104 | <0.0001 |
| GLZLM_LZE | 69.5 | <0.0001 | <0.0001 | <0.0001 | 1 | 0.1601 | 0.0432 | <0.0001 | <0.0001 | <0.0001 | <0.0001 | <0.0001 | <0.0001 | <0.0001 | 0.0218 | 0.0046 | 1 |
| GLZLM_LGZE | 110.5 | <0.0001 | <0.0001 | <0.0001 | <0.0001 | <0.0001 | <0.0001 | 1 | 0.0295 | 1 | <0.0001 | 1 | 0.0586 | 0.0008 | 0.0002 | 0.1292 | <0.0001 |
| GLZLM_HGZE | 28.6 | <0.0001 | <0.0001 | <0.0001 | <0.0001 | <0.0001 | 1 | 1 | 1 | 0.1437 | <0.0001 | 1 | 0.0469 | <0.0001 | 0.2697 | <0.0001 | <0.0001 |
| GLZLM_SZLGE | 83.3 | <0.0001 | <0.0001 | <0.0001 | <0.0001 | <0.0001 | <0.0001 | 1 | 0.0052 | 1 | <0.0001 | 0.671 | 1 | 0.0002 | 0.0063 | 0.2458 | <0.0001 |
| GLZLM_SZHGE | 17.8 | <0.0001 | 0.0023 | 0.0192 | 0.0013 | <0.0001 | 1 | 1 | 1 | 0.0002 | 0.004 | 1 | <0.0001 | 0.0315 | 0.0005 | 0.0023 | <0.0001 |
| GLZLM_LZLGE | 146.5 | <0.0001 | <0.0001 | <0.0001 | 1 | 0.1981 | 0.0189 | 1 | <0.0001 | <0.0001 | <0.0001 | <0.0001 | <0.0001 | <0.0001 | 1 | 1 | 1 |
| GLZLM_LZHGE | 36.7 | <0.0001 | 1 | <0.0001 | 1 | 0.0238 | 0.136 | <0.0001 | 1 | 0.0015 | 0.0122 | <0.0001 | <0.0001 | <0.0001 | 0.0388 | 0.2063 | 1 |
| GLZLM_GLNU | 10.4 | <0.0001 | 1 | <0.0001 | 1 | <0.0001 | 0.0603 | 0.0001 | 1 | 0.0013 | 1 | 0.0003 | 1 | 0.0696 | 0.0042 | 1 | 0.4545 |
| GLZLM_ZLNU | 78.4 | <0.0001 | <0.0001 | <0.0001 | <0.0001 | <0.0001 | <0.0001 | 1 | 0.1256 | 1 | 1 | 0.5066 | 1 | 0.9184 | 1 | 0.001 | 0.0856 |
| GLZLM_ZP | 95.7 | <0.0001 | <0.0001 | <0.0001 | <0.0001 | <0.0001 | <0.0001 | <0.0001 | 1 | <0.0001 | 1 | 0.0003 | <0.0001 | <0.0001 | <0.0001 | 1 | <0.0001 |

**NOTE**. Post-hoc tests corresponded to Tukey tests with Bonferroni corrections for multiple comparisons.

**Supplementary Data 5.** Coefficients attributed to the radiomics features from the final elasticnet models, depending on the use of intensity harmonization techniques (IHT). The last column corresponds to the number of time a radiomics feature was selected over the 6 final models.

| **Radiomics features** | **Coefficients** | | | | | | |
| --- | --- | --- | --- | --- | --- | --- | --- |
|  | **IHT_fat_** | **IHT_std_** | **IHT_HM.1_** | **IHT_HM.All_** | **IHT_HM.AllC_** | **No-IHT** | **No. of selections** |
| **HISTO_min** | -0.19708586 | 0 |  | 0 | 0 | 0 | 1 |
| **HISTO_mean** |  | 0 | -0.00936217 | -0.01185317 | 0 | 0 | 2 |
| **HISTO_std** | 0.152781415 | 0 |  | 0 | 0 | 0 | 1 |
| **HISTO_max** |  | 0 |  | 0 | 0 | 0 | 0 |
| **HISTO_Q1** | -0.2153927 | -0.0547508 | -0.12849343 | -0.0847394 | -0.0271987 | 0 | 5 |
| **HISTO_Q2** | 0 | 0 | 0 | -0.02300587 | -0.00456617 | 0 | 2 |
| **HISTO_Q3** | 0 | 0 | 0 | 0 | 0 | 0 | 0 |
| **HISTO_Skewness** | 0.456433612 | 0.285224366 | 0 | 0 | 0 | 6.16E-05 | 3 |
| **HISTO_Kurtosis** | 0 | 0 | 0 | 0 | 0 | 0 | 0 |
| **HISTO_ExcessKurtosis** | 0 | 0 | 0 | 0 | 0 | 0 | 0 |
| **HISTO_Entropy_log10** | 0.180358062 | 0 | 0 | 0 | 0 | 0 | 1 |
| **HISTO_Entropy_log2** | 0.18053796 | 0 | 0 | 0 | 0 | 0 | 1 |
| **HISTO_Energy** |  | 0 | 0 | 0 | 0 | 0 | 0 |
| **GLCM_Homogeneity** |  | 0 | 0 | 0 | 0 | 0 | 0 |
| **GLCM_Energy** |  | -0.04378031 | 0 | 0 | 0 | 0 | 1 |
| **GLCM_Contrast** | -0.3123872 | -0.15254472 | 0 | 0 | 0 | 0 | 2 |
| **GLCM_Correlation** | 0.112292974 | 0.124708138 | 0 | 0 | 0 | 0.004544154 | 3 |
| **GLCM_Entropy_log10** | 0 | 0 | 0 | 0 | 0 | 0 | 0 |
| **GLCM_Entropy_log2** | 0 | 0 | 0 | 0 | 0 | 0 | 0 |
| **GLCM_Dissimilarity** | -0.1152313 | 0 | 0 | 0 | 0 | 0 | 1 |
| **GLRLM_SRE** | 0 | 0 | 0 | 0 | 0 | 0 | 0 |
| **GLRLM_LRE** | -0.01753752 | 0 | 0 | 0 | 0 | 0 | 1 |
| **GLRLM_LGRE** | 0.14348966 | 0 | 0 | 0 | 0 | 0 | 1 |
| **GLRLM_HGRE** | 0 | -0.0803369 | -0.03942021 | -0.00118087 | 0 | 0 | 3 |
| **GLRLM_SRLGE** | 0.11218717 | 0 | 0 | 0 | 0 | 0 | 1 |
| **GLRLM_SRHGE** | 0 | -0.0961111 | -0.03609806 | 0 | 0 | 0 | 2 |
| **GLRLM_LRLGE** | 0 | 0 | 0.10711252 | 0.040962633 | 0.018302967 | 0 | 3 |
| **GLRLM_LRHGE** | 0 | 0 | 0 | 0 | 0 | 0 | 0 |
| **GLRLM_GLNU** | 0 | 0.005647922 | 0 | 0 | 0 | 0 | 1 |
| **GLRLM_RLNU** | 0 | 0 | 0 | 0 | 0 | 0 | 0 |
| **GLRLM_RP** | 0 | 0 | 0 | 0 | 0 | 0 | 0 |
| **NGLDM_Coarseness** | 0 | -0.0478834 | 0 | 0 | 0 | 0 | 1 |
| **NGLDM_Contrast** | -0.1443031 | 0 | 0 | 0 | 0 | 0 | 1 |
| **NGLDM_Busyness** | 0.03050319 | 0.198655742 | 0 | 0 | 0 | 0 | 2 |
| **GLZLM_SZE** | 0 | 0 | 0 | 0 | 0 | 0 | 0 |
| **GLZLM_LZE** | 0.26971496 | -1.1754694 | 0 | 0 | 0 | 0 | 2 |
| **GLZLM_LGZE** | 0 | 0 | 0 | 0 | 0 | 0 | 0 |
| **GLZLM_HGZE** | 0 | -0.02011805 | -0.0099492 | 0 | 0 | 0 | 2 |
| **GLZLM_SZLGE** | 0 | 0 | 0 | 0 | 0 | 0 | 0 |
| **GLZLM_SZHGE** | -0.0031098 | -0.08296594 | -0.0663328 | 0 | 0 | -0.07386323 | 4 |
| **GLZLM_LZLGE** | 0.129648178 | -7.23865899 | 0 | 0 | 0 | 0 | 2 |
| **GLZLM_LZHGE** | 0.28467021 | 0 | 0 | 0 | 0 | 0 | 1 |
| **GLZLM_GLNU** | 0.232078778 | 0.009991263 | 0 | 0 | 0 | 0 | 2 |
| **GLZLM_ZLNU** | 0.043217124 | 0 | 0 | 0 | 0 | 0 | 1 |
| **GLZLM_ZP** | -2.45158692 | -0.8152943 | 0 | 0 | 0 | 0 | 2 |
| SHAPE_Volume | 0 | 0 | 0 | 0 | 0 | 0 | 0 |
| SHAPE_Sphericity | -1.97127657 | 0 | 0 | 0 | 0 | 0 | 1 |
| SHAPE_Compacity | 0 | 0.01614248 | 0.00311782 | 0 | 0 | 0 | 2 |
